# A conserved arginine in NS5 binds genomic 3′ stem–loop RNA for primer-independent initiation of flavivirus RNA replication

**DOI:** 10.1261/rna.078949.121

**Published:** 2022-02

**Authors:** Sai Wang, Kitti Wing Ki Chan, Min Jie Alvin Tan, Charlotte Flory, Dahai Luo, Julian Lescar, Jade K. Forwood, Subhash G. Vasudevan

**Affiliations:** 1 Program in Emerging Infectious Diseases, Duke-NUS Medical School, 169857 Singapore; 2 Lee Kong Chian School of Medicine, Nanyang Technological University, 636921 Singapore; 3 School of Biological Sciences, Nanyang Technological University, 637551 Singapore; 4 School of Biomedical Sciences, Charles Sturt University, Wagga Wagga, New South Wales 2650, Australia; 5 Department of Microbiology and Immunology, National University of Singapore, 117545 Singapore; 6 Institute for Glycomics, Griffith University, Gold Coast Campus, QLD 4222, Australia

**Keywords:** de novo initiation, Dengue virus 3' stem–loop, Dengue NS5 protein, Dengue virus, flaviral RNA replication

## Abstract

The commitment to replicate the RNA genome of flaviviruses without a primer involves RNA–protein interactions that have been shown to include the recognition of the stem–loop A (SLA) in the 5′ untranslated region (UTR) by the nonstructural protein NS5. We show that DENV2 NS5 arginine 888, located within the carboxy-terminal 18 residues, is completely conserved in all flaviviruses and interacts specifically with the top-loop of 3′SL in the 3′UTR which contains the pentanucleotide 5′-CACAG-3′ previously shown to be critical for flavivirus RNA replication. We present virological and biochemical data showing the importance of this Arg 888 in virus viability and de novo initiation of RNA polymerase activity in vitro. Based on our binding studies, we hypothesize that ternary complex formation of NS5 with 3′SL, followed by dimerization, leads to the formation of the de novo initiation complex that could be regulated by the reversible zipping and unzipping of *cis*-acting RNA elements.

## INTRODUCTION

Dengue remains a major public health issue in more than 100 countries where two-thirds of the world's population resides. The disease is caused by the dengue virus (DENV), which is transmitted by the *Aedes* mosquito. The four DENV serotypes (DENV 1–4) along with Zika virus (ZIKV), Yellow fever virus (YFV), West Nile virus (WNV), tick-borne encephalitis virus (TBEV), and the zoonotic Usutu virus (USUV) are members of the *flavivirus* genus belonging to the family *Flaviviridae* (Gubler 1998). Despite millions of infection cases each year, there are no specific antivirals against DENV or any flaviviruses. Hence there is an urgent need for therapeutic candidates against these viruses that are partly driven by vaccine rollout issues and other well-publicized limitations of approved vaccines such as Dengvaxia (Thomas and Yoon 2019).

The ∼11k nucleotide (nt) positive-strand RNA genome of DENV contains a long open reading frame (ORF) that encodes a polyprotein that is post-translationally cleaved by viral and host proteases into three structural proteins (capsid, envelope, and membrane) and seven nonstructural proteins (NS1, NS2A, NS2B, NS3, NS4A, NS4B, and NS5). The ORF is flanked by 5′ and 3′ untranslated regions (UTR) that are 100 and 450 nt long, respectively, which contain multiple secondary structure elements (Ng et al. 2017; Barrows et al. 2018). The two conserved stem–loop structures SLA and SLB in 5′UTR are required for viral RNA replication, with SLA acting as a promoter that is believed to have a large binding footprint on NS5 (Filomatori et al. 2006, 2011; Lodeiro et al. 2009; Lee et al. 2021). SLB contains the sequence element known as 5′ UAR (upstream of ATG region) that is complementary to a sequence within the 3′UTR involved in genome circularization and is flanked by the AU-rich region that has been suggested to form a *cis*-acting conformationally tuned RNA element termed 5′UAR-flanking stem (UFS) (Liu et al. 2016). The secondary structures in 3′UTR can be divided into three domains (Alvarez et al. 2005a). The first two domains contain stem–loop structures SLI and SLII and dumbbell structures DB1 and DB2 (Chapman et al. 2014; Clarke et al. 2015; Akiyama et al. 2016; Slonchak and Khromykh 2018). The 3′SL structure within Domain III of 3′UTR is highly conserved among flaviviruses and contains sequences that have been shown to interact with the DENV RNA-dependent RNA polymerase (RdRp) in a yeast three-hybrid assay (Hodge et al. 2016), as well as being important for WNV replication but not translation (Tilgner et al. 2005). In addition to the secondary structures, there are three pairs of complementary sequences (CS, UAR, DAR) from 5′ and 3′ regions of the genome, which together with other functionally important long-range RNA–RNA interactions within the ORF region, drive genome circularization for initiation of replication (Filomatori et al. 2006; Villordo and Gamarnik 2009).

NS5 is the largest and most conserved DENV protein and is the key player in viral RNA replication. It is composed of two enzymatic domains, the amino-terminal methyltransferase (MTase) and carboxy-terminal RNA-dependent RNA polymerase (RdRp) (Dong et al. 2012; Saw et al. 2015), and can also suppress host innate antiviral response (Ashour et al. 2009). The dynamic intramolecular interaction between the two NS5 domains is facilitated by the interdomain linker region, which is critical for DENV replication (Zhao et al. 2015a,b; Subramanian Manimekalai et al. 2016). MTase domain catalyzes methylation of the viral genome 5′ cap structure to form a type 1 cap, which ensures the stability and integrity of the genome (Chang et al. 2016). RdRp adopts a canonical right-hand conformation consisting of three subdomains, thumb, fingers, and palm, which carry out processive RNA synthesis. DENV RNA replication occurs within vesicle-like structures formed by the invagination of a rough endoplasmic reticulum (ER) membrane within which the replication complex (RC) consisting of viral nonstructural proteins as well as unknown host proteins orchestrate RNA replication (Welsch et al. 2009; Klema et al. 2015; Brand et al. 2017; Lescar et al. 2018). Recently it was shown that the 3′SL is required for the formation of ER-derived vesicle packets in DENV infected cells (Cerikan et al. 2020). Although NS5's role in RNA replication is carried out within these membrane-restricted vesicles in the cytoplasm (Welsch et al. 2009), a large proportion of the protein is found in the nucleus of DENV2 infected cells (Pryor et al. 2007; Hannemann et al. 2013; Kumar et al. 2013; Tay et al. 2013). The carboxy-terminal 18 (C_ter_18) residues (882–900) of DENV2 NS5 contain a nuclear localization signal (NLS) sufficient for its recognition as a cargo by the host importin α nuclear transport protein for translocation to the nucleus (Tay et al. 2016). Although the NLS function can be disrupted without affecting viral RNA synthesis by mutation of residues required for importin binding, mutation of R888, a residue completely conserved in all flaviviruses leads to nonviable DENV2 (Tay et al. 2013). In addition, NS5 dimerization has been observed in proteins from both DENV and ZIKV and could be required for the formation of a cooperative complex of MTase and RdRp, enabling capping of newly synthesized RNA emerging from the RdRp in trans by the MTase domain from the other monomer (Klema et al. 2016; Saw et al. 2019). The C_ter_18 region is often not resolved in crystal structures of flaviviral NS5 (Yap et al. 2007; Zhao et al. 2015a), except in the case where NS5 dimers were observed and the C_ter_18 region contained an α helix that protruded from the NS5 molecule into the MTase domain of the interacting NS5 partner (Klema et al. 2016). Here, R888 was found to be within H-bonding distance to Y838 of the same monomer which is also completely conserved in all the major flavivirus phylogenetic groups (Supplemental Fig. 1) and has been identified as a potential RNA binding residue (Hodge et al. 2016).

It is widely accepted that recruitment of NS5 by 5′SLA acting as a promoter initiates negative-strand RNA synthesis (Lodeiro et al. 2009; Bujalowski et al. 2020). In contrast, how the SLA-recruited-NS5 translocates over to the 3′ end to initiate primer-independent RNA synthesis on the 3′ end of the (−) RNA strand remains an enigma. Here, we used reverse genetics to show that R888 and the neighboring Y838 residue are critical for viable virus production, and we used RNA binding assays with NS5 and various NS5 mutants to identify a key structural interaction for the formation of a de novo RNA initiation complex between residue R888 at the NS5 carboxy-terminal tail and the top-loop of 3′SL to establish specific interactions. We propose a mechanistic model for flavivirus replication consistent with the most recent structural and biological studies of NS5 and its RNA ligands.

## RESULTS

### Carboxy-terminals Y838 and R888 play a critical role in DENV2 replication

**FIGURE 1.**
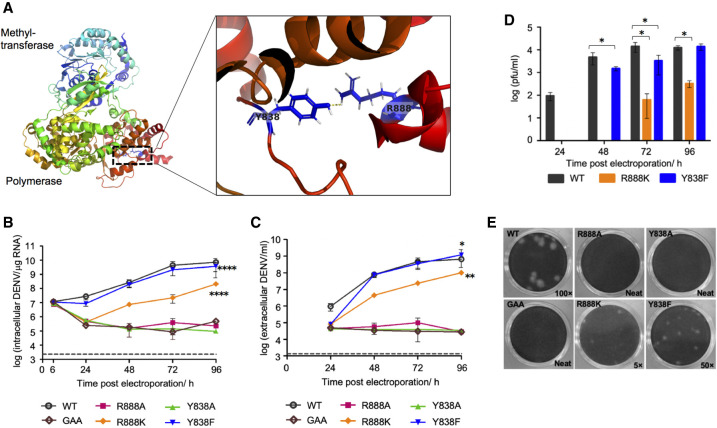
Mutational analysis of NS5 carboxy-terminal residues Y838 and R888 in DENV2 by reverse genetics. (*A*) 3D structure of a DENV3 NS5 monomer (PDB access code: 5CCV.A [Klema et al. 2016]) with an enlarged view of the NS5 carboxy-terminal region showing hydrogen bond formation between Tyrosine 838 and Arginine 888 through their side chains. Replication characteristics of DENV2 WT NS5 and NS5 mutants are shown in *B*–*E*. BHK-21 cells were electroporated with 10 µg of DENV2 WT or mutant infectious clone RNA and the replication kinetics was followed until 96 h post-transfection. The replication-deficient NS5 mutant GAA was included as negative control. (*B*) Real-time PCR quantification of intracellular viral RNA at the indicated timepoints. The dotted line represented the detection level for mock-transfected cells. (*C*) Real-time PCR quantification of extracellular viral RNA in the supernatants of the transfected cells at the indicated timepoints. The dotted line represented the detection level in the supernatant of mock-transfected cells. (*D*) Virus titer in the supernatants of transfected BHK-21 cells measured by plaque assay. (*E*) Pictures showing plaque morphologies of DENV2 WT and NS5 mutant viruses. The dilution at which plaques can be observed is indicated. The data in *B*–*D* are presented as average ± SD from two independent experiments. Differences in intracellular (*B*) and extracellular (*C*) viral RNA kinetics between groups were compared by two-way ANOVA with Bonferroni correction. Mean values of the virus titers (*D*) between WT and the respective mutants are compared by unpaired Student's *t*-test, and a *P*-value <0.05 was considered significant (*) *P* < 0.05, (**) *P* < 0.01, (****) *P* < 0.0001.

**FIGURE 2.**
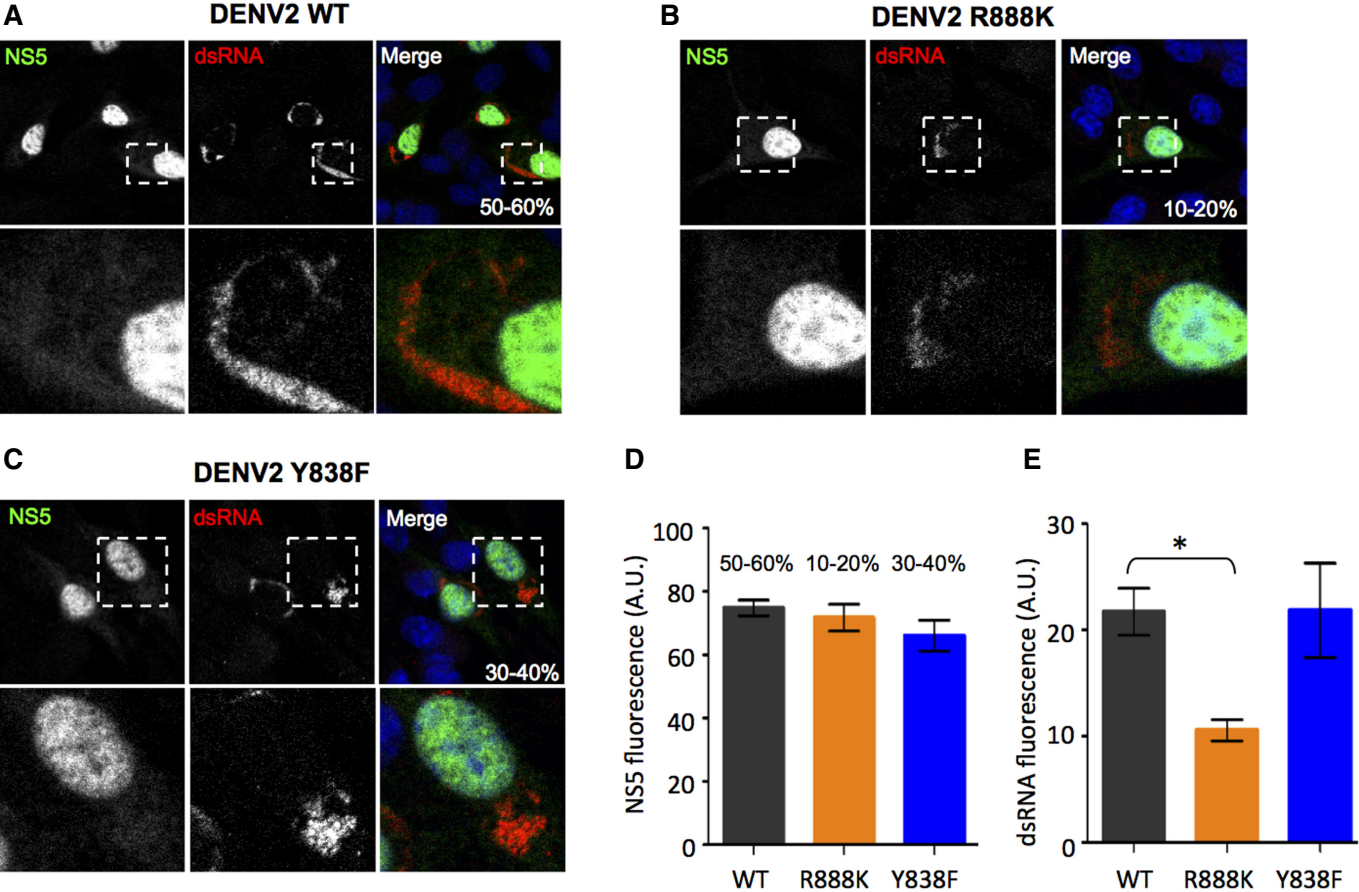
Subcellular distribution of NS5 and dsRNA in WT and mutant DENV transfected cells. BHK-21 cells were transfected with DENV2 WT (*A*), R888K (*B*), and Y838F (*C*) mutants as in Figure 1B and analyzed at 72 h post-transfection for NS5 (green) and dsRNA (red) by immunofluorescence assay. Digitized images were captured by Zeiss LSM710 upright confocal microscope using a 63× oil immersion lens. The % infectivity is indicated, and the *insets* in the *bottom* panes show the zoom-in views of the dotted box regions. Two independent experiments were performed, and the representative images were observed for more than 99% of the cells. Intensity quantification of (*D*) NS5 and (*E*) dsRNA fluorescence of WT, R888K, and Y838F infected cells. Data are presented as bar graphs with average ± SD obtained from *n* = 30 infected cells (nine field images for WT, 24 field images for R888K, and 15 field images for Y838F). Values indicated on *top* of the bars of graph in *D* are the % infectivity of the respective viruses. Difference in fluorescence intensity between WT and R888K or Y838F was compared by Student's *t*-test and a *P*-value <0.05 was considered significant (*) *P* < 0.05.

We previously identified the carboxy-terminal 18 residues of DENV2 NS5 as a NLS recognized by host importins to transport the NS5 protein to the nucleus of infected cells. Arginine 888 forms the P3 residue of the NS5 NLS binding determinant for importin α (Tay et al. 2016). During evolution, this arginine residue has been strictly conserved in NS5 amino acid sequences from the four major flavivirus phylogenetic groups: mosquito-borne (MBFV), tick-borne (TBFV), insect-specific (ISFV), and no known vector (NKV) flaviviruses (Supplemental Fig. 1). Residue R888 interacts with residue Y838 (Fig. 1A; Supplemental Fig. 1) in one of the molecules in the unit cell of DENV3 NS5 crystal structure where the carboxy-terminal end is ordered via the formation of a dimeric interaction (Klema et al. 2016), while it is not observed in other structures because Cter18 is disordered (Yap et al. 2007; Zhao et al. 2015a). To uncover any functional importance of this interaction, we introduced site-specific mutations into a DENV2 cDNA clone and examined their impact on virus replication and subcellular localization of NS5. Four single mutations Y838A, Y838F, R888A, or R888K were introduced individually into the DENV2 cDNA clone by site-directed mutagenesis (Tay et al. 2015). The infectious clone RNA containing each of these four mutations was generated by in vitro transcription (IVT) and transfected into BHK-21 cells to examine the replication kinetics over 96 h. The replication-deficient NS5 polymerase active site mutant “G_662_DD” to “GAA” (referred to as GAA mutant) was included as negative control. BHK-21 cells transfected with Y838F IVT RNA showed approximately two- to threefold lower viral RNA synthesis (*P* < 0.0001) and extracellular RNA (*P* = 0.019) levels to that of wild-type (WT) (Fig. 1B,C). However, the infectious virus was detected from 48 h post-transfection onward with an approximately threefold lower infectious titer as compared to WT at 48 h (*P* = 0.045) and 72 h post-transfection (*P* = 0.042) (Fig. 1D), suggesting a lower infectivity of the resultant Y838F virus. This is supported by the observation of smaller plaque size (Fig. 1E) and lower percentage infection of Y838F (30%–40%) at 72 h post-transfection compared to WT (50%–60%) (Fig. 2). On the other hand, R888K mutant exhibited severe attenuation in virus replication, with ∼2 log lower genome synthesis (*P* < 0.0001), extracellular RNA (*P* = 0.004), and infectious virus production (*P* = 0.023 at 72 h; *P* = 0.017 at 96 h post-transfection) compared to WT (Fig. 1B–D). This slower replication kinetics of R888K is consistent with its smaller plaque phenotype compared to WT (Fig. 1E) and low percentage infection (10%–20%) at 72 h post-transfection (Fig. 2B). Similar to the replication-defective GAA mutant, both Y838A and R888A mutants showed no increase in the viral RNA synthesis and extracellular RNA over 96 h (Fig. 1B,C), which is consistent with the lack of any plaque detection even without any dilution (Fig. 1E), indicating that these mutations are lethal to the virus. Collectively, these results suggest that mutations at these conserved positions of NS5 had an impact on the virus replication.

We next examined the NS5 subcellular localization of its Y838F, R888K mutants in comparison to WT NS5 by confocal microscopy. In agreement with a previous report (Tay et al. 2016), NS5 of WT DENV2 was found mainly in the nucleus with a small fraction in the cytoplasm that colocalized with dsRNA to carry out genome replication (Fig. 2A). Neither Y838F nor R888K mutations altered NS5 nuclear localization (Fig. 2B,C). Interestingly, although the NS5 mutants (R888K and Y838F) have lower percentage infectivity compared to WT, the NS5 fluorescence intensity of these mutants was comparable to WT (Fig. 2D). Notably, the R888K mutant exhibited a significantly lower double-stranded (ds) RNA staining intensity compared to the WT (*P* = 0.03; Fig. 2E). This observation is consistent with the lower replication (Fig. 1B) and infectivity (Fig. 2B) of the R888K mutant, suggesting that the conserved arginine at position 888 may play a crucial role in viral RNA synthesis.

Overall, our data show that NS5 carboxy-terminal residues Y838 and R888 play a critical role in DENV2 replication independent of R888's role as a determinant of NS5 subcellular localization (Tay et al. 2016).

### NS5 Y838 and R888 play a critical role in RNA polymerase activities

**FIGURE 3.**
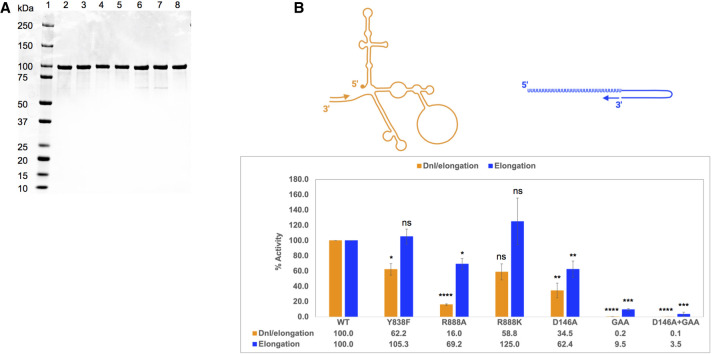
Assessment of primer-independent de novo initiation/elongation and primer-dependent elongation polymerase activities of WT NS5 and mutant. (*A*) SDS-PAGE analysis of WT NS5 and NS5 mutant recombinant proteins expressed in *E. coli* and purified by His-tag affinity and size exclusion chromatography. Samples from left to right are (1) protein ladder, (2) WT, (3) Y838F, (4) R888A, (5) R888K, (6) D146A, (7) GAA, (8) D146A + GAA. (*B*) The cartoon depicts de novo initiation/elongation on the DENV 2 mini-replicon (Zhao et al. 2015b) (*left*) and elongation from a primed template (*right*). The de novo initiation/elongation and elongation polymerase activities of NS5 mutants Y838F, R888A, R888K, D146A, GAA, and D146A + GAA are compared with WT NS5 which was normalized to 100%. The data is presented as average ± SD from two to four independent repeat experiments. The statistical significance of difference between the two groups was evaluated by Student's *t*-test, and a *P*-value <0.05 was considered significant (≤0.0001, ≤0.001, ≤0.01, ≤0.05 were represented by [****], [***], [**], [*], respectively).

In order to explore a potential role of residues Y838 and R888 in viral RNA replication, the polymerase activity of the NS5 single mutants Y838F, R888A, and R888K was examined by de novo initiation/elongation (referred to as dni/elongation) and elongation assays in vitro (Fig. 3B; Zhao et al. 2015a,c). The recombinant DENV2 NS5 WT and mutant proteins generated by site-directed mutagenesis were expressed in *Escherichia coli* (Fig. 3A). The NS5 Y838A mutant was unstable and could not be purified, which could account for this mutation being lethal in our transfection studies (Fig. 1B,C,E). The enzymatic activity of each mutant NS5 protein was normalized against that of WT NS5 that was set as 100% (Fig. 3B). Compared to WT, the dni/elongation polymerase activity of purified NS5 Y838F and R888K was reduced by ∼40% which may be attributed to the breakdown of the hydrogen bond between the side chains of the two residues. On the other hand, the elongation activity of Y838F mutant protein was similar to WT, and that of R888K was slightly stimulated compared to WT. The NS5 R888A mutant protein resulted in a dramatic reduction in the dni/elongation polymerase activity by more than 80% compared to WT NS5, while the elongation activity was lowered by 30%, suggesting that R888 may play a crucial role in de novo RNA initiation.

Since NS5 is a multifunctional protein where the amino-terminal MTase domain has been shown to modulate the carboxy-terminal RdRp and vice versa (Potisopon et al. 2014; Zhao et al. 2015b), we included the NS5 D146A (part of the MTase “KDKE” active site [Zhao et al. 2015c]) MTase defective mutant protein, the G_662_DD → GAA RdRp defective mutant protein, and a combined D146A + GAA mutant in the polymerase activity assays. The MTase inactive D146A mutation led to a significant decrease in both dni/elongation and primer-dependent elongation activities of NS5 polymerase by ∼65% and 40%, respectively, suggesting that MTase is in cross-talk with the RdRp activity through intramolecular signaling between the two domains which could potentially influence NS5's affinity for RNA substrate. The polymerase inactive GAA mutation completely abolished the dni/elongation activity as expected; however, it still retained ∼10% of the elongation activity in vitro. The D146A + GAA mutant completely abolished both dni/elongation and elongation activities. Taken together the in vitro polymerase assays indicate that the NS5 residues Y838 and R888 play a role in RNA replication.

### Carboxy-terminal R888 modulates NS5 binding affinity with 5′SLA and 3′SL

**FIGURE 4.**
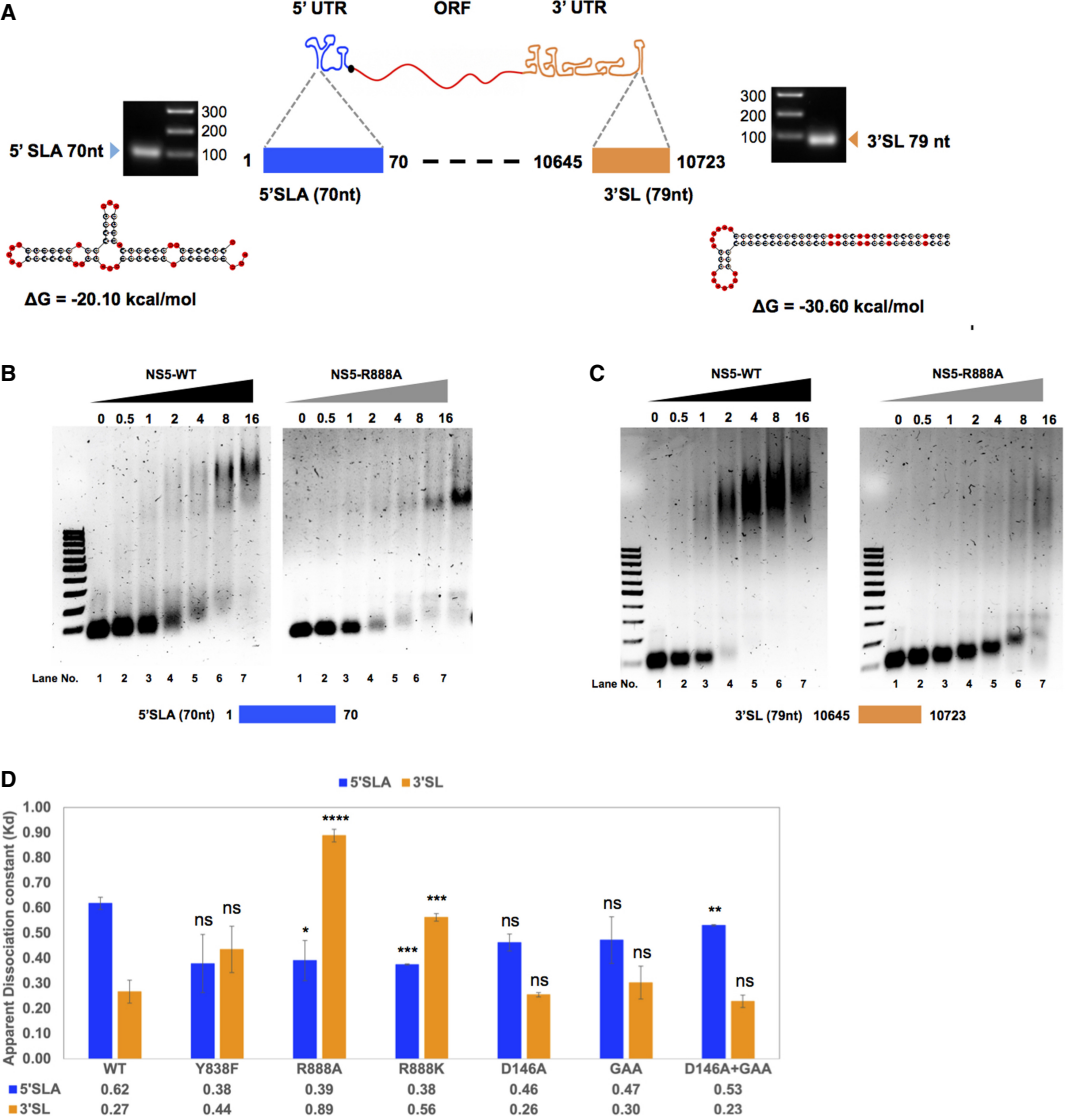
Analysis of DENV2 NS5 binding to 5′ SLA or 3′SL RNA. (*A*) Schematic illustration of DENV2 genome showing the position, sequence, and size of 5′ terminal stem–loop A (5′ SLA) and 3′ terminal stem–loop (3′SL) within the 5′ and 3′ untranslated regions (UTRs). The purity of the in vitro synthesized and purified 5′ SLA and 3′SL RNA samples were analyzed by agarose gel electrophoresis. Secondary structures of 5′SLA and 3′SL were predicted by mfold web server (Zuker 2003) with the thermodynamic free energy ΔG of the folding process at 37°C indicated. (*B*,*C*) RNA electrophoretic mobility shift assay (REMSA) for 5′ SLA (B) or 3′ SL (C) with WT NS5 or NS5 R888A mutant, analyzed on 1.2% agarose gel with lane number indicated at the *bottom*. Lane *1* in each of the four gels represented 5′ SLA (*B*) or 3′ SL (*C*) RNA alone in binding buffer. Lanes *2*–*7* represented binding reactions of NS5 with RNA at molar ratios of 0:1, 0.5:1, 1:1, 2:1, 4:1, 8:1, and 16:1 as indicated on *top* of each lane. (*D*) Binding affinity of 5′ SLA or 3′ SL with WT NS5 or NS5 mutants Y838F, R888A, D146A, GAA, D146A + GAA reflected by apparent dissociation constant (*K*_d_) was calculated from band intensities using ImageJ from two to four independent repeat experiments represented as average ± SD. The statistical significance of difference between the two groups was evaluated by Student's *t*-test and a *P*-value <0.05 was considered significant (≤0.0001, ≤0.001, ≤0.01, ≤0.05 were represented by [****], [***], [**], [*], respectively).

The precise role of Y838 and R888 of NS5 is not known but the dni/elongation polymerase activity assay results suggest that they play a role in RNA binding events associated with viral RNA replication. To examine this further, we investigated the interactions of the WT and mutant NS5 proteins with the conserved RNA secondary structural features in the 5′UTR and 3′-UTR regions focusing on the 70 nt 5′SLA and 79 nt 3′SL, respectively (Fig. 4A). The secondary structures for the 5′ SLA and 3′SL were predicted using the mfold web server (Zuker 2003), and the thermodynamic negative free energy of the folding process at 37°C suggests that the RNAs used in our study are folded into a stable structure.

5′SLA and 3′SL RNA were generated by in vitro transcription as described previously. The purity of RNA samples was analyzed by agarose gel electrophoresis as shown in Figure 4A. The RNA electrophoretic mobility shift assay (REMSA) was conducted to assess protein–RNA binding. Purified WT or mutant NS5 was mixed with a fixed amount of 5′SLA or 3′SL RNA at molar ratios ranging from 0:1, 0.5:1, 1:1, 2:1, 4:1, 8:1, and 16:1, followed by electrophoresis in agarose gels and staining with GelRed for RNA visualization as shown in Figure 4B,C. Samples of 0:1 molar ratio of NS5 and RNA mixture were loaded on lane 1 of each gel showing RNA alone in the binding buffer (50 mM HEPES pH 7.0, 150 mM NaCl, 10% glycerol, 1 mM TCEP, and 0.5 mg/mL BSA). As the amount of NS5 was increased in the binding reaction, the intensity of the free RNA band at the bottom reduced, as the RNA binds to NS5 to form a complex that migrates slower. Taking the WT NS5 and 3′SL binding reactions as an example (Fig. 4C, left panel), the slow migrating NS5–RNA complex was faintly visible for the 0.5:1 ratio. At a 1:1 ratio, the free RNA intensity was reduced by around 40% and the larger band started to appear as a broad smear. The intensity of the larger band was more prominent from a 2:1 molar ratio of NS5 to RNA. To quantify the binding affinity, the size and band intensity of free RNA remaining for each reaction ratio were quantified with ImageJ software (Schindelin et al. 2015). The RNA binding affinity was calculated for WT NS5 and each of the mutants with 5′SLA and 3′SL and presented as an apparent dissociation constant (*K*_d_) in Figure 4D where tighter affinity corresponds to a smaller *K*_d_ value. The *K*_d_ value of WT NS5 with 5′SLA is in the micromolar range and is in a similar *K*_d_ range measured for ZIKV full-length NS5 binding with ZIKV 5′SLA (Bujalowski et al. 2020). Mutation of residue R888 to either A or K resulted in a slightly higher binding affinity of NS5 with 5′SLA in comparison with WT, bearing in mind that REMSA is a semiquantitative technique. Remarkably, NS5 R888A and R888K mutant proteins showed reduced binding affinity with 3′SL by over three- and twofold, respectively compared to WT NS5. The NS5 Y838F mutation showed no significant change in binding with 5′SLA or 3′SL, again probably due to the lack of sensitivity of REMSA. The binding of MTase and RdRp active sites mutants (D146A, GAA or D146A + GAA) with 5′SLA and 3′SL were similar to WT, suggesting that the defective enzymatic functions were not directly linked to NS5 binding interactions with 5′SLA and 3′SL. This may have implications for the recent finding that the 3′ terminal sequence of DENV genome has a nonreplicative role in the formation of membranous replications organelles (Cerikan et al. 2020). When we incubated the WT and R888A NS5 proteins with yeast tRNA, we did not detect an appreciable interaction between yeast tRNA and both NS5 proteins (Supplemental Fig. 2B). In addition, the addition of yeast tRNA at vast molar excess (>100-fold) did not affect WT NS5's ability to bind 3′SL. Taken together, the binding we demonstrate between NS5 and 3′SL is specific and dependent on the R888 residue.

### 5′SLA-bound NS5 complex has an affinity for the 3′SL-bound NS5

**FIGURE 5.**
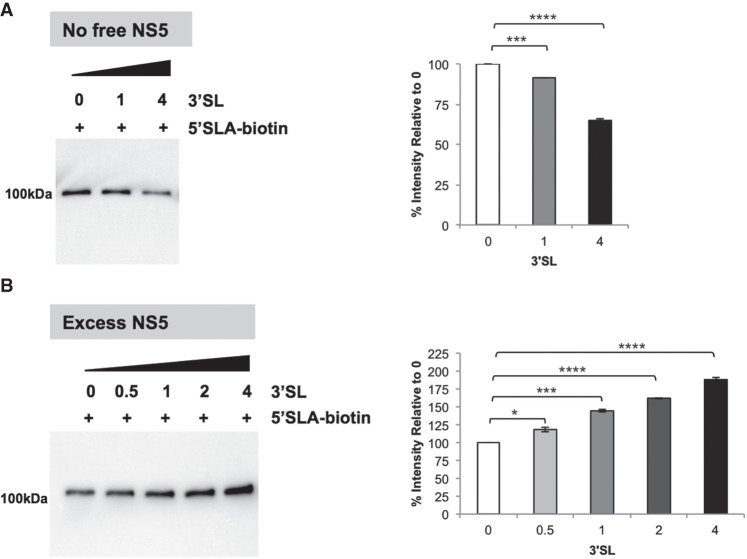
Western blot analysis of WT NS5 binding to biotinylated 5′SLA RNA following coimmunoprecipitation. (*A*) WT NS5 was incubated with immobilized biotinylated 5′SLA. After excess unbound NS5 was removed, increasing amounts of 3′SL were added (*left*). Quantitation of the NS5 band was performed using ImageJ and normalized to 100% for the sample with no added 3′SL (*right*). (*B*) WT NS5 was incubated with immobilized biotinylated 5′SLA as in *A*, but increasing amounts of 3′SL were added without removal of excess unbound NS5 (*left*). Quantitation of the NS5 was done as in (*A*) (*right*). Molar ratio of 3′SL to 5′SLA is shown on *top* of the gel image, and NS5 was detected using the 5M1 antibody. The statistical significance of the difference between two groups was evaluated by Student's *t*-test and a *P*-value <0.05 was considered significant (≤0.0001, ≤0.001, ≤0.01, ≤0.05 were represented by [****], [***], [**], [*], respectively).

In order to examine the interactions of 5′SLA and 3′SL in NS5 in solution using coimmunoprecipitation, we labeled 5′SLA with biotinylated cytidine by adding it to its 3′ end with T4 RNA ligase. The biotinylated 5′SLA was immobilized onto the streptavidin Sepharose beads and incubated with NS5 at a 2:1 molar ratio of NS5 to 5′SLA to form the 5′SLA–NS5 complex. We found that removal of excess NS5 by stringent washing followed by the addition of various amounts (molar ratio of 3′SL to 5′SLA ranges from 0:1 to 4:1) of unlabeled 3′SL resulted in a significant decrease of SLA-bound NS5 at high 3′SL concentration (*P* < 0.0001) (Fig. 5A). Interestingly, if 3′SL is added directly to the 5′SLA–NS5 mixture without the washing step to remove the excess NS5, we detect an increase in NS5 binding with increasing amounts of 3′SL (Fig. 5B) (*P* < 0.05), suggesting an additional association of NS5 to the 5′SLA attached beads facilitated by 3′SL most likely in the form of a 3′SL–NS5 complex.

Taken together, the coimmunoprecipitation data suggests 3′SL can displace NS5 that is bound to immobilized 5′SLA when the free NS5 amount is limited. However, in conditions where excess free NS5 is present together with 3′SL, a more intricate NS5–RNA complex forms that needs further investigation.

### Carboxy-terminal R888 is critical for the formation of NS5–3′SL complex

**FIGURE 6.**
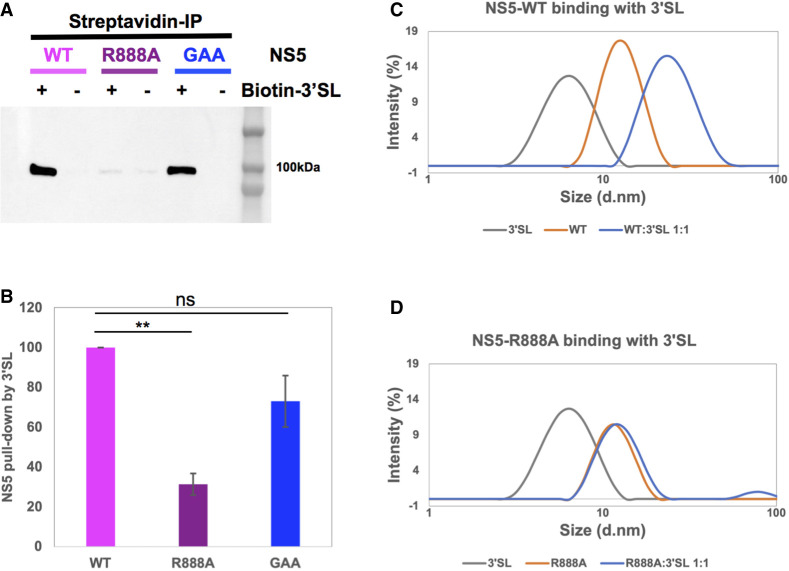
Analysis of 3′SL RNA interaction with WT and mutant NS5 by coimmunoprecipitation and dynamic light scattering. (*A*) Western blot analysis showing pull-down of WT NS5, R888A mutant, and GAA mutant through 3′SL RNA immobilized on streptavidin Sepharose beads or the “no-RNA” mock coupled Sepharose beads as control. NS5 was detected by in-house 5M1 antibody (ref) which recognizes the MTase domain. (*B*) Quantification of pull-down WT, R888A, and GAA with the amount of WT normalized to 100%. The data were represented as average ± SD from two independent repeat experiments. The statistical significance of difference between the two groups was evaluated by Student's *t*-test and a *P*-value <0.05 was considered significant (≤0.0001, ≤0.001, ≤0.01, ≤0.05 were represented by [****], [***], [**], [*], respectively). (*C*,*D*) Intensity-based size distribution profiles from dynamic light scattering (DLS) experiments showing 3′SL RNA, NS5 WT, or molar ratio 1:1 mixture of NS5 and 3′SL complex (*C*) or R888A mutant (*D*) and the molar ratio 1:1 mixture of NS5 R888 and 3′SL. The DLS experiments were repeated twice with similar size distribution profiles for each sample.

After establishing that R888 may be crucial for dni/elongation of polymerase and also demonstrating that it dramatically decreased binding affinity for 3′SL, we carried out further coimmunoprecipitation studies to assess complex formation between the NS5 R888A mutant and 3′SL. A biotinylated cytidine was added to the 3′ end of the 79 nt 3′SL RNA molecule with T4 RNA ligase in order to immobilize it on the streptavidin Sepharose beads. Binding of NS5 WT, R888A, or GAA to the immobilized 3′SL was assessed after stringent wash steps to remove nonspecific binding. The NS5 proteins bound to the beads were then analyzed by SDS-PAGE followed by western blot detection using in-house NS5 5M1 antibody (Fig. 6A; Zhao et al. 2014). Direct binding of NS5 or mutants to mock coupled Sepharose beads (labeled as “no-RNA”) was carried out as control in parallel. The western blot showed that WT NS5 was pulled down by immobilized 3′SL specifically, suggesting that the RNA secondary structure was sufficiently exposed and available for NS5 binding in solution. The polymerase-inactive GAA mutant bound similarly implying that 3′SL binding occurred independently of the polymerase active site mutations consistent with the small increase in polymerase elongation assay in vitro (see above). In contrast, a >70% reduction in pull-down was observed for the R888A mutant (Fig. 6B) showing that the arginine residue was a major determinant of NS5 recognition of the 3′SL. The presence of the R888A mutant in the supernatant after centrifugation following the incubation with beads (Supplemental Fig. 2) demonstrated that the single alanine substitution did not impact NS5 stability or solubility.

To further evaluate the stable binding of NS5 to 3′SL in solution and compare it with the R888A mutant, dynamic light scattering (DLS) and gel filtration analysis of the protein–RNA complex were carried out. Figure 6C,D shows the intensity-weighted size distribution profiles of the particles present in samples of NS5 (WT or R888A), 3′SL, and the mixture of NS5 and 3′SL with a 1:1 molar ratio generated from the intensity profile of the scattered light at 25°C. A 1:1 mixture of WT NS5 and 3′SL sample contained larger particles than WT NS5 or 3′SL alone (Fig. 6C), suggesting the formation of WT NS5–3′SL complex. In contrast, the size distribution curve of the mixture of R888A with 3′SL (Fig. 6D) almost overlapped with that of the R888A alone, indicating an absence in the formation of the R888A NS5–3′SL complex. Since the scattering intensity is determined by the sixth power of the size of molecules (Stetefeld et al. 2016), the intensity-weighted size distribution curve of R888A (∼97%) and 3′SL (∼3%) mixture mainly represented the larger molecule R888A. To ensure a reliable estimation of particle size, WT NS5, R888A mutant, and 3′SL RNA samples were measured twice at two different concentrations to eliminate the concentration effect (Supplemental Fig. 3; Stetefeld 2016).

Taken together, our results from Co-IP and DLS suggested that the R888A mutation abolished the formation of the NS5–3′SL complex.

### NS5 R888 and the top-loop of 3′SL are the key players in NS5–3′SL interaction

**FIGURE 7.**
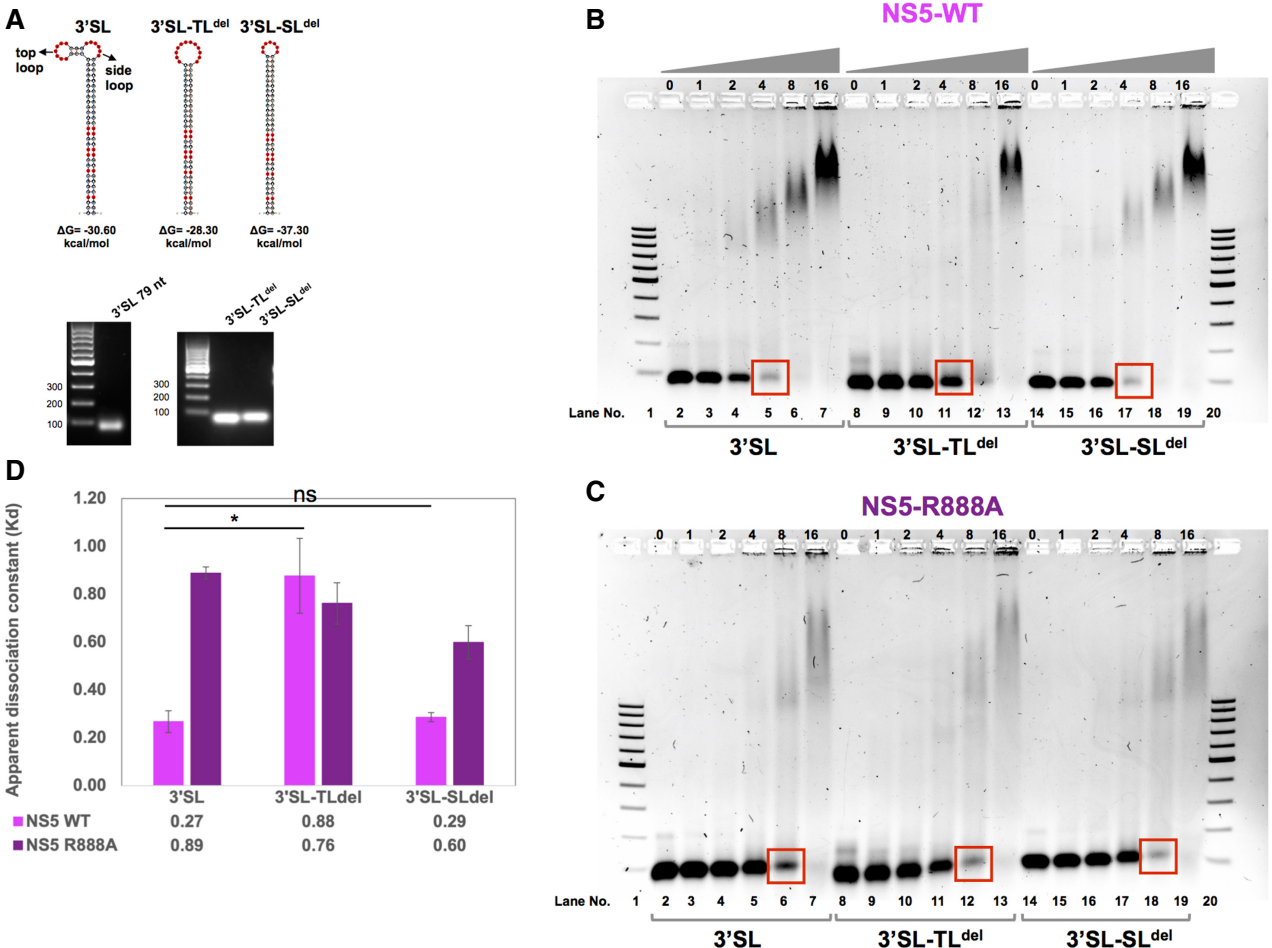
Analysis of DENV2 NS5 binding to 3′SL RNA and 3′SL RNA with top-loop or side-loop deletion. (*A*) Secondary structures of 3′SL RNA and 3′SL with deleted top lop (3′SL-TL^del^) or side-loop (3′SL-SL^del^) were predicted by mfold web server with folding free energy ΔG at 37°C shown *below*. Corresponding RNA samples produced by in vitro transcription followed by purification were analyzed by agarose gel electrophoresis. (*B*,*C*) RNA electrophoretic mobility shift assay (REMSA) for NS5 WT (*B*) or R888A mutant (*C*) with 3′ SL, 3′SL-TL^del^, or 3′SL-SL^del^ analyzed on 1.2% agarose gel with lane number indicated at the *bottom*. Lanes *2*, *8*, and *15* in both gels represented 3′SL, 3′SL-TL^del^, 3′SL-SL^del^ RNA alone in binding buffer. Lanes *3*–*7*, *9*–*13*, and *15*–*19* represented binding reactions of NS5 with RNA at molar ratios of 0:1, 1:1, 2:1, 4:1, 8:1, and 16:1 as indicated on *top* of each lane. The REMSA experiments were performed three times with similar results. (*D*) Binding affinity of WT NS5 or R888A mutant with 3′ SL, 3′SL-TL^del^, or 3′SL-SL^del^ reflected by apparent dissociation constant (*K*_d_) was calculated from three independent repeat experiments represented as average ± SD. The statistical significance of difference between the two groups was evaluated by Student's *t*-test and a *P*-value <0.05 was considered significant (≤0.0001, ≤0.001, ≤0.01, ≤0.05 were represented by [****], [***], [**], [*], respectively).

We next investigated whether the NS5–3′SL interaction is mediated by a specific sequence/structure within the 3′SL. The secondary structure of 3′SL (Fig. 7A) as predicted by mfold (Zuker 2003) is composed of a long stem, a top-loop, and a side-loop. The flavivirus-conserved sequence 5′-(C)ACAG-3′ in the top-loop of 3′SL has been shown to be essential for WNV RNA synthesis (Tilgner et al. 2005). In DENV, the 5′-ACAG-3′ sequence has been suggested to interact with RdRp (Hodge et al. 2016). Hence, we examined whether R888 in DENV2 NS5, which is conserved among four major groups of flaviviruses (MBFV, TBFV, ISFV, NKV), is involved in the recognition of the top-loop in 3′SL. Two 3′SL deletion mutants were generated, to delete either the top-loop (3′SL-TL^del^) or the side-loop (3′SL-SL^del^) of 3′SL. The mfold (Zuker 2003) prediction for the loop-deleted RNA is shown in Figure 7A. RNA samples of 3′SL and its deletion mutants were produced by IVT as described previously (Wang et al. 2020). The purity of RNA samples was analyzed by agarose gel electrophoresis as shown in Figure 7A.

The binding interactions of WT NS5 and R888A mutant with 3′SL, 3′SL-TL^del^, and 3′SL-SL^del^ were assessed by REMSA as previously described. Mixtures of NS5 and RNA with molar ratios ranging from 0:1, 1:1, 2:1, 4:1, 8:1, and 16:1 were assessed by agarose gel electrophoresis followed by RNA visualization (Fig. 7B,C). WT NS5 binding to the 3′SL-SL^del^ was similar to that of 3′SL, as both 3′SL (lane 5) and 3′SL-SL^del^ (lane 17) were almost depleted at a 4:1 molar ratio suggesting the formation of a complex with WT NS5 (Fig. 7C). In contrast, the majority of 3′SL-TL^del^ still remained as free RNA in lane 11. Correspondingly, the apparent dissociation constant *K*_d_ values (Fig. 7D) suggested that deletion of the side-loop from 3′SL had almost no effect on its binding affinity with WT NS5, while deletion of the top-loop led to a dramatic reduction in the binding affinity with NS5 by over threefold. In contrast, neither the top-loop nor the side-loop had an impact on the binding of 3′SL with the R888A mutant as shown by the similar RNA band-shift patterns (Fig. 7C) and the comparable *K*_d_ values (Fig. 7D) among all three RNAs.

Taken together, NS5 residue R888 that forms part of the C_ter_18 NLS is essential for the DENV2 virus life cycle due to its crucial role in de novo RNA synthesis modulated by the binding interaction with the top-loop region of 3′SL.

## DISCUSSION

We previously discovered that the carboxy-terminal 18 (C_ter_18) residues of DENV NS5 protein played a crucial role in RNA replication, but the precise mechanism remained unclear at that time (Tay et al. 2016). Within C_ter_18, amino acids 886-MKRFR-890 of DENV2 NS5 were determined by crystallography to be the binding region (P1–P5) to host importin α nuclear transport protein (Tay et al. 2016). Introduction of the R888A mutation into a DENV2 infectious clone was lethal, while the R888K mutant was severely attenuated even though it was still localized in the nucleus (Fig. 2). We therefore hypothesized that the DENV2 NS5 residue R888, which is completely conserved among the four major flaviviral phylogenetic groups (MBFV, TBFV, ISFV, NKV), plays a crucial role in RNA replication (Supplemental Fig. 1B). The carboxy-terminal region of NS5 is often not observed in NS5 crystal structures as it is considered to be a highly dynamic part of the protein located within the thumb subdomain (Zhao et al. 2015a). The high mobility/dynamics of the thumb subdomain was also shown previously by hydrogen-deuterium exchange mass spectrometry and consistent with the higher B factor of the region in the crystal structure (Zhao et al. 2015a). We found that the alanine mutation of Y838 was lethal to the virus although the Y838F was only slightly attenuated in agreement with its potential RNA binding role. Unexpectedly, the NS5 Y838A recombinant protein could not be expressed in *E. coli* while the Y838F mutant protein was readily purified for biochemical studies. It is possible that the interaction of Y838 with a structured region of the viral genomic RNA may assist in its folding, although this needs to be experimentally evaluated in the future. However, purified NS5 proteins carrying the mutations at R888A and Y838F were significantly affected (R888A > Y838F) in de novo initiation/elongation polymerase activity compared with primer-dependent polymerase elongation activity (Fig. 3). These virological and in vitro biochemical assays pointed to an important mechanistic role for R888 in binding to a specific RNA structural element during replication. The REMSA binding studies showed that the R888A mutation led to a very significant loss of binding to the 3′SL element in comparison to the Y838F, D146A (MTase deficient mutant), GAA (RdRp deficient mutant), or the D146 + GAA mutations. In an in vitro biochemical setting, the R888 binding to 3′SL is not dependent on the polymerase active site since the RdRp deficient NS5 GAA mutant and WT are pulled-down similarly by 3′SL immobilized on beads, while R888A mutant protein pull-down is very much diminished. Taken together, the dramatic decrease in the R888A mutant's binding affinity with 3′SL demonstrated by REMSA, RNA immunoprecipitation, and DLS analysis, and its >80% decrease in de novo initiation/elongation polymerase activity suggest a critical role of R888 in viral replication through specific interactions with 3′SL. Furthermore, deletion of the top-loop from the 3′SL structure predicted by mfold (Zuker 2003) resulted in a drastic decrease in NS5 binding affinity with 3′SL, in agreement with the finding that the deletion of pentanucleotide 5′-CACAG-3′ in the analogous region of the 3′UTR region of WNV was essential for RNA replication but not required for translation (Tilgner et al. 2005). From these studies, we can posit that the pentanucleotide within the top-loop is recognized by NS5 through specific protein–RNA interactions.

So, what is the crucial function of R888 in flaviviral RNA replication? The flaviviral genome contains highly structured UTRs and complementary sequences at 5′ and 3′ ends that promote genome circularization for replication (Alvarez et al. 2005a,b; Filomatori et al. 2006, 2011; Clyde et al. 2008; Lodeiro et al. 2009) and numerous dynamic long-range RNA interactions >4k bases apart within the ORF of the genome that appear to be functionally important (Huber et al. 2019). The 5′SLA is the promoter and key element for flaviviral genome replication (Lodeiro et al. 2009; Filomatori et al. 2011). The NS5 binding to 5′SLA together with the genome cyclization sequences juxtaposes the 3′ end of the viral RNA genome adjacent to the SLA-bound NS5 for NS5's translocation to the 3′ end for de novo primer-independent RNA synthesis. However, there is a gap in our knowledge about the atomic details of how NS5 specifically binds to SLA and/or how it translocates to the 3′ end to initiate de novo RNA replication. Studies with ZIKV NS5 suggest that both the thumb subdomain and the MTase region are involved in binding SLA (Bujalowski et al. 2020) which potentially could permit the translocation of SLA-bound NS5 to the 3′ end for de novo initiation and transcription of the new RNA strand. Interestingly the 5′ promoter element binding to NS5 has been further refined by the finding that the 5′UTR includes a conformationally tuned *cis*-acting AU-rich RNA element called 5′ UAR flanking stem (UFS) that is critical for NS5 recruitment and replication (Liu et al. 2016). The model proposed by Liu et al. (2016) does not consider how a 5′ SLA-bound NS5 can translocate to the 3′ end for de novo initiation and RNA genome replication. While other RNA viruses have evolved specific mechanisms for this crucial step (Appleby et al. 2015), there is no evidence for sequence-specific RNA interactions to account for flaviviral de novo initiation in the published literature.

**FIGURE 8.**
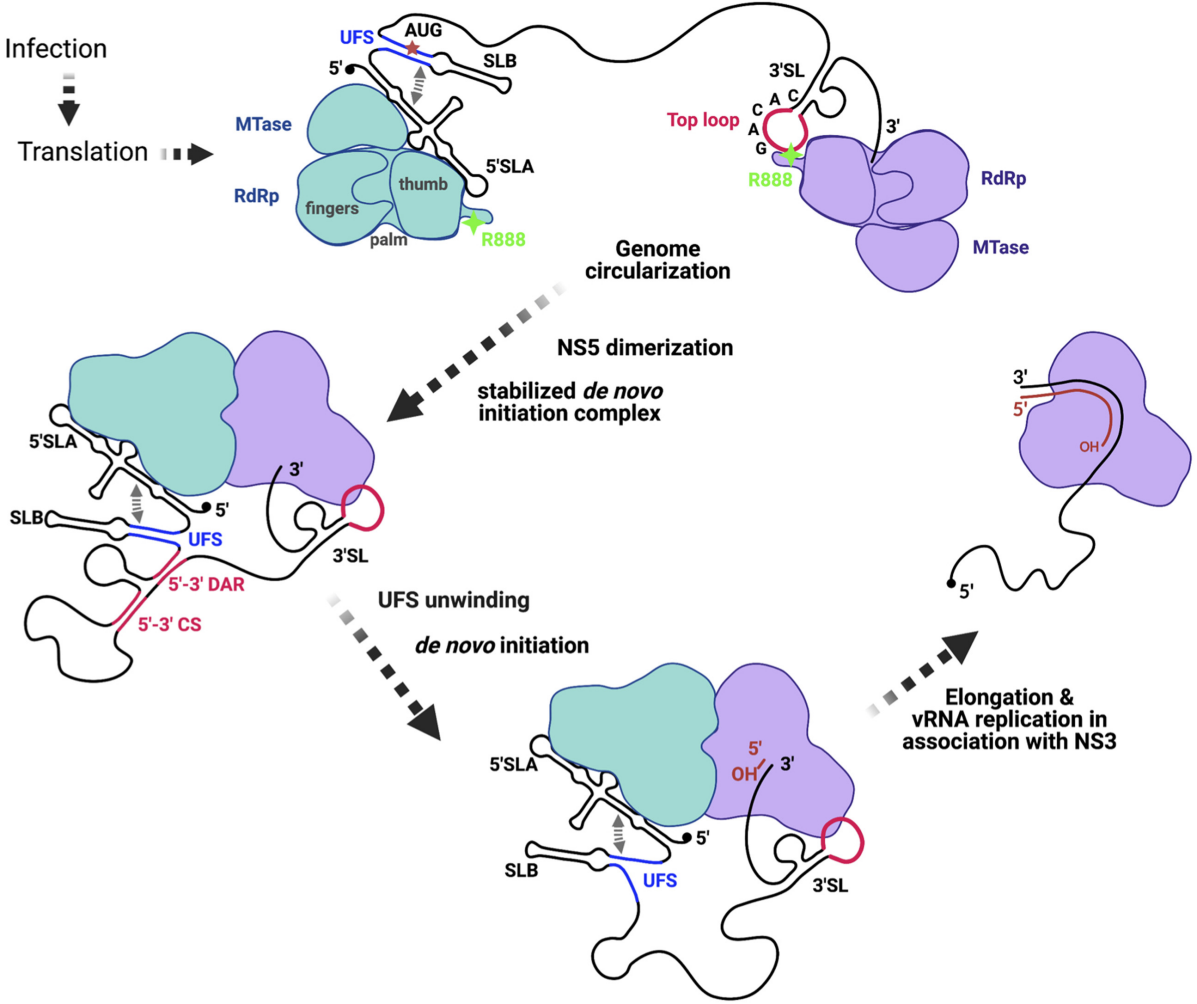
A mechanistic model of de novo initiation and elongation of flavivirus genomic RNA, highlighting the critical events of genome circularization, NS5 dimerization, and UFS-mediated de novo initiation and elongation (see text for details). NS5 (green) bound to 5′ UTR (SLA and SLB) is well established by Gamarnik group (Filomatori et al. 2006, 2011; Lodeiro et al. 2009) and the present study showed the specific interaction between a molecule of NS5 (purple) through R888 at its carboxy-terminal region and the top-loop of 3′SL. Long-range RNA interactions within the open reading frame region (Huber et al. 2019) juxtaposes the 5′ and 3′ CS and DAR for RNA cyclization supported by NS5–RNA complex formation. The *cis*-acting tunable UFS region ensures primer-independent RNA replication by serving as a gate keeper to ensure the formation of a productive stable de novo initiation complex that can carry out processive RNA replication in concert with viral and host proteins.

In hepatitis C virus (HCV), a *Flaviviridae* family member, the carboxyl terminus of the RdRp and the β-loop are thought to occlude the active site and the entry of RNA template as well as incoming and priming nucleotides. The dynamics of the carboxyl terminus and gate keeping by the β-loop enable the formation of a de novo initiation complex that undergoes conformational transition to attain a processive elongation state (Appleby et al. 2015). Based on our demonstration of sequence-specific RNA binding of NS5 R888 to 3′SL (Fig. 8), we propose the following scenario. (1) Once the viral genomic RNA extends and serves as a template for translation by host machinery, sufficient polyproteins are synthesized that are post-translationally cleaved by viral and host proteases. The resulting NS5 proteins bind at the 5′ UTR via SLA (Filomatori et al. 2006, 2011; Lodeiro et al. 2009) stabilized by UFS (which also includes SLB) (Supplemental Fig. 4) and at the 3′UTR via the top-loop of 3′SL (this study). Translation will be blocked by 5′ UTR–NS5 interaction (this includes SLA and SLB) (Liu et al. 2016) thus permitting the genome circularization via extensive long-range RNA interactions (Huber et al. 2019), the 5′ and 3′ cyclization and DAR sequences (Alvarez et al. 2005b). (2) Next, the NS5 molecules bound to the 5′ and 3′ RNA structures associate into a larger complex as supported by our observation that NS5 loaded on to sepharose bead with immobilized 5′ SLA binds more of the excess NS5 when free 3′SL is added in a dose-dependent manner (Fig. 5). As mentioned, the DENV NS5 dimerization was first observed in the crystal structure obtained by Choi and colleagues (Klema et al. 2016) which supports our observation of increased NS5 binding in our RNA pull-down experiment (Fig. 7B). (3) As a multidomain protein where both the MTase and RdRp domains of NS5 are in constant intramolecular communication (Potisopon et al. 2014; Zhao et al. 2015b; Bujalowski et al. 2020), we posit that the two NS5 molecules will undergo conformational stabilization that positions the template 3′ end and incoming nucleotides within proximity of the active site. (4) Distinct from the proposal by Liu et al. (2016), we hypothesize that the zipping and unzipping of the AU-rich UFS may provide the crucial thermodynamic energy for the formation of the stable de novo initiation complex. Liu et al. (2016) elegantly showed through reverse genetics that both disrupting and stabilizing the base-pairing of UFS which are completely conserved in all flaviviruses were lethal. The AU-rich sequence is therefore most probably energetically favored to zip and unzip to permit the 3′SL bound NS5 to add the incoming nucleotide to the dinucleotide primer (Selisko et al. 2012) in the de novo initiation process that could occur through defined steps that remain unknown without the necessary RNA–protein structures. (5) Once the de novo initiation steps are completed, the 3′SL bound NS5 interacts with NS3 (Johansson et al. 2001; Brooks et al. 2002; Zou et al. 2011; Tay et al. 2015) and enters processive RNA replication.

Overall, we demonstrate in this work for the first time that DENV2 NS5 residue R888 which is conserved in all flaviviruses specifically recognizes the 3′SL top-loop, which is essential for de novo initiation of genome replication. We also propose a highly plausible model depicting the formation of a stabilized *dni* complex mediated by both 5′SLA and 3′SL RNA that requires further biophysical validation that is beyond the scope of the current study. Given the highly conserved property of both R888 and 3′SL, our findings also pave the way toward the development of a pan-flavivirus therapeutic solution targeting the critical de novo initiation step in RNA replication.

## MATERIALS AND METHODS

### Cells

BHK-21 cells (baby hamster kidney fibroblast cells; ATCC) were maintained in RPMI 1640 medium (Gibco) supplemented with 10% [v/v] fetal bovine serum (FBS) and 1% [v/v] penicillin–streptomycin (P/S) at 37°C with 5% CO_2_.

### DENV infectious clone generation

Full-length DENV2-3295 infectious cDNA clone (GenBank accession: EU081177) used in this study has been previously described (Tay et al. 2015). pWSK29 D2 fragment 3 (Tay et al. 2015, 2016) was used to introduce the respective NS5 mutations (Y838F, Y838A, R888K, R888A, and G662DD → GAA) by a QuikChange II XL Site-Directed Mutagenesis Kit (Agilent Technologies) following the product manual. Primer sequences used here are available upon request. Sequences of the resulting mutants were confirmed by a DNA sequencing service provided by first BASE. Fragment 3 containing the desired mutations was excised from the vector by XbaI and SacI and cloned into pWSK29 D2 fragment 1 + 2 plasmid that was similarly cut with XbaI and SacI.

### Transfection and in vitro virus replication assay

The full-length cDNA clones (WT and NS5 mutants) were linearized by SacI and purified using the phenol-chloroform method. RNA was then in vitro transcribed from the linearized plasmid using the T7 mMESSAGE mMACHINE Kit (Ambion). The in vitro transcribed RNA was transfected into BHK-21 cells using the previously described electroporation conditions to examine the replication profile of the viruses over 96 h (Tay et al. 2015). Supernatants were collected at indicated time-points for plaque quantification by standard BHK-21 plaque assay and extracellular viral RNA by real-time RT-PCR. Cells were then washed once with PBS prior to lysing with TRIzol for quantification of intracellular viral RNA by real-time RT-PCR.

### RNA extraction and real-time RT-qPCR

For extracellular viral gRNA quantification, viral RNA from the supernatants was extracted by the QIAamp Viral RNA Extraction Kit (Qiagen) according to the manufacturer's instructions. Viral load in the supernatants was obtained by real-time RT-qPCR in Bio-Rad real-time thermal cycler CFX96 using iTaq Universal SYBR Green One-Step Kit (Bio-Rad) using primers 5′-CAGGCTATGGCACTGTCACGAT-3′ and 5′-CCATTTGCAGCAACACCATCTC-3′ targeting the DENV2 envelope protein region (Wang et al. 2020). Absolute viral RNA genome copy was calculated based on the standard curve generated from the in vitro RNA product of the DENV2-3295 E gene and reported as genome copy per mL of supernatant (Paradkar et al. 2011; Tay et al. 2015).

Total RNA was isolated from cell lysates using the TRIzol (Invitrogen) extraction method and subjected to cDNA synthesis using the ImProm-II Reverse Transcription System (Promega) according to manufacturer's instructions. Quantification of intracellular viral gRNA was conducted by real-time PCR using iQ SYBR Green Supermix (Bio-Rad). PCR product of DENV2-3295 E gene was used to generate the standard curve for quantification of viral genome copy. The values of intracellular viral RNA genome copies were normalized to actin expression and reported as genome copy per µg of total RNA.

### Plaque assay

A total of 2 × 10^5^ BHK-21 cells were seeded into a 24-well plate and incubated overnight at 37°C in 5% CO_2_. Viral supernatants were serially diluted with serum-free RPMI 1640 media and inoculated onto the cell monolayer for 1 h. Virus inoculums were then removed, and cells were overlaid with 0.8% carboxylmethyl cellulose (CMC) and further incubated for 4–5 d. Infected cells were fixed with 3.7% formaldehyde and stained with 1% crystal violet for plaque visualization.

### Immunofluorescence assay

Transfected cells on coverslips were fixed with ice-cold methanol at −20°C for 15 min. Antibodies against DENV NS5 (human monoclonal IgG clone, 5R3 [Zhao et al. 2014] and dsRNA [Scicons]) were used for detection of the viral antigens. Digitized images were captured using a Zeiss LSM710 upright confocal microscope (Carl Zeiss) at 64× magnification. Image processing and fluorescence intensity quantification were done with ImageJ software (Collins 2007).

### RNA production

#### 5′SLA and 3′SL RNA

The 70 nt-long 5′SLA (5′-AGTTGTTAGTCTACGTGGACCGACAAAGACAGATTCTTTGAGGAAGCTAAGCTTAACGTAGTTCTAACAG-3′) and 79 nt-long 3′SL (5′-AGATCCTGCTGTCTCCTCAGCATCATTCCAGGCACAGAACGCCAGAAAATGGAATGGTGCTGTTGAATCAACAGGTTCT-3′) RNAs were generated by in vitro transcription using the MEGAscript Kit (Life Technologies) following the product manual. The DNA templates were PCR amplified from a minireplicon construct followed by purification by gel extraction using the QIAquick Gel Extraction Kit. The synthesized RNAs were purified by the lithium chloride precipitation method following the MEGAscript Kit (Life Technologies) manual, followed by analysis on a 1.5% agarose gel with 1× TAE buffer under 100V for 1 h. GelRed (Biotium) was added into agarose at 1:10,000 v/v right before gel casting for detection of RNA using the Chemidoc Imaging system.

#### DENV2 mini-replicon RNA as template for polymerase de novo initiation/elongation assay

A minireplicon RNA containing 5′ and 3′-UTR and part of the capsid coding sequence (Supplemental Fig. 5A) was used as a template for the in vitro polymerase de novo initiation/elongation assay. The minireplicon RNA was synthesized and capped with m^7^G(5′)ppp(5′)A cap structure analog (New England Biolabs) by in vitro transcription using the MEGAscript Kit (Life Technologies) following the product manual. RNA purification and agarose gel electrophoresis were carried out as described above.

#### 3′SL RNA with top-loop (3′SL-TL^del^) or side-loop (3′SL-SL^del^) deletion

Deletion of sequences for top-loop (7 nt 5′-ACAGAAC-3′) or side-loop (6 nt 5′-AGAAAA-3′) from a template DNA construct which is part of the 3′UTR was done by overlap PCR. After successful construction of 3′SL-TL^del^ and 3′SL-SL^del^ DNA templates, RNA synthesis and purification were done as mentioned above.

### RNA biotinylation

Biotinylation of the purified 5′SLA and 3′SL RNA was carried out using the Thermo Scientific Pierce RNA 3′ End Biotinylation Kit. A single biotinylated cytidine (bis)phosphate nucleotide was ligated to the 3′ terminus of the single RNA strand by T4 RNA ligase followed by purification steps to remove the enzyme and other components that remained in the ligation reaction according to the product manual.

### Prediction of RNA secondary structure

The secondary structures for the 5′ SLA and 3′SL were predicted with mfold web server (Zuker 2003) and the thermodynamic free energy of the folding process at 37°C was calculated based on the nearest-neighbor energetics and further refined by the efn2 program based on coaxial stacking (Doshi et al. 2004).

### Site-directed mutagenesis

The Y838A, Y838F, R888A, R888K, D146A, G662DD → GAA, and D146A + GAA mutations on DENV2 NS5 were done using the QuikChange II XL Site-Directed Mutagenesis Kit (Agilent Technologies) following the product manual. Primer sequences used here are available upon request. Sequences of the resulting mutants were confirmed by DNA sequencing service provided by first BASE.

### Expression and purification of DENV2 recombinant WT NS5 and mutants

The NS5 coding sequence was constructed in pProEX-HTb vector with an amino-terminal His6 tag followed by a TEV cleavage site. Expression and purification of the recombinant full-length NS5 of DENV2 have been described previously (Zhao et al. 2015a; Wang et al. 2020). *E. coli* cells were resuspended in His Buffer A (50 mM Tris-HCl pH 7.5, 500 mM NaCl, 10% glycerol, 10 mM β-mercaptoethanol, 10 mM imidazole) and lysed with 0.1 volume of 10× FastBreak Cell Lysis Reagent (Promega), followed by DNase treatment and clarified by centrifugation. The supernatant was filtered by a 0.45-µm syringe filter unit and loaded onto a HisTrapFF column and eluted by a linear gradient of imidazole from 10 to 500 mM. Fractions containing His-tagged protein were bagged into a SnakeSkin (Thermo Scientific, 30 k MWCO) membrane tubing together with TEV for His tag cleavage and concomitantly dialyzed against His buffer A at 4°C for 18 h or overnight. The cleavage mixture was reloaded onto the HisTrapFF column to separate any possible un-cleaved NS5 protein. The flow-through sample from the second run of His affinity chromatography was concentrated and loaded onto a HiPrep 26/60 Sephacryl S-200 HR column (GE Healthcare Life Sciences) which was equilibrated in S200 Buffer (20 mM HEPES pH 7.5, 300 mM NaCl, 10% glycerol, 2 mM DTT) for further purification by size exclusion chromatography. The purity of elution fractions was confirmed by SDS-PAGE.

### NS5 in vitro activity assay

Both the de novo initiation/elongation assay (also called primer-independent polymerase assay) and the elongation assay (also called primer-dependent polymerase assay) have been described previously (Zhao et al. 2015a; Tay et al. 2016). Briefly, the de novo initiation/elongation assay reaction consists of 100 nM DENV2 NS5, 100 nM minireplicon RNA (with sequence and features shown in Supplemental Fig. 5A), 20 µM ATP, 20 µM GTP, 20 µM UTP, 5 µM BBT-CTP (Jena Bioscience), in a buffer containing 50 mM Tris-HCl, pH 7.5, 10 mM KCl, 1 mM MgCl_2_, 0.3 mM MnCl_2_, 0.001% Triton X-100, and 10 µM cysteine. The elongation assay reaction consists of 100 nM DENV2 NS5, 100 nM poly(U) RNA template as shown in Supplemental Figure 5B, 3 µM BBT-ATP (Jena Bioscience), in a buffer containing 50 mM Tris-HCl, pH 7.5, 10 mM KCl, 0.5 mM MnCl_2_, 0.001% Triton X-100, and 10 µM cysteine.

Both assay reactions were incubated at 37°C for exactly 1 h, terminated by addition of 10 µL 2.5× STOP buffer (200 mM NaCl, 25 mM MgCl_2_, 1.5 M DEA, pH 10) supplemented with 25-nM calf intestinal alkaline phosphatase (CIP, New England Biolabs) to remove the pyrophosphate (PPi) from the BBT group. The amount of fluorescent BBT group was measured from a 384-well flat-bottom, black plate (Corning) by the Tecan Spark 10M microplate reader at excitation and emission wavelengths of 422 and 566 nm, respectively. Each tested condition was recorded from triplicate reactions.

### RNA–protein complex formation and RNA electrophoretic mobility shift assay

The protocol of RNA electrophoretic mobility shift assay (REMSA) has been described previously (Wang et al. 2020). Briefly, a set of RNA–NS5 binding interactions were set up in binding buffer (150 mM NaCl, 1 mM TCEP, 10% glycerol, 0.5 mg/mL BSA, 50 mM HEPES pH 7.0) with the molar ratio of NS5 to RNA ranging from 0:1 to 16:1 in a total of 25 µL, in which the RNA was fixed at 160 nM and NS5 varied from 0 to 2.56 µM. The binding reactions were seated on ice for 20 min and loaded to agarose gel run under 100 V for 100 min. GelRed (Biotium) was added into the agarose at 1:10,000 v/v right before gel casting for RNA and RNA–protein complex detection using a Chemidoc Imaging system (Bio-Rad) with adjusted exposure time to avoid oversaturation of the RNA bands. Formation of the RNA–protein complex was identified based on the retardation of RNA on the native agarose gel. Band intensity of the unbound free RNA was quantified by ImageJ. The amount of NS5-bound RNA was calculated by subtracting the amount of unbound RNA from the total RNA input. Percentage of NS5-bound RNA was plotted against NS5 concentration, from which the apparent *K*_d_ value was calculated by GraphPad Prism 5 using the equation

$$Y=B_{\max}*X^{h}/K_{d}^{h}+X^{h},$$

where Y is the percentage of NS5-bound RNA, B_max_ is the maximum specific binding in the same unit as Y, X is the concentration of NS5 protein, h is the Hill slope defining the cooperativity of the binding interaction, *K*_d_ is the protein concentration required to achieve half-maximum binding at equilibrium, expressed in the same unit as X.

### RNA–NS5 coimmunoprecipitation (Co-IP)

An amount of 25 pmole of biotinylated RNA was added to 100 µL of streptavidin beads (GE Healthcare Streptavidin HP) preequilibrated in buffer 0 (150 mM NaCl, 50 mM Tris, pH 7.5) and incubated at 4°C for 30 min with gentle rotation followed by blocking with 2 mM biotin in buffer 0. An amount of 25 pmole of WT NS5 or mutant was added to the RNA tagged beads which were reequilibrated with buffer 1 (150 mM NaCl, 1 mM TCEP, 0.5 mg/mL BSA, 1% Triton X-100, 50 mM Tris, pH 7.5) and incubated for 1 h with gentle rotation at 4°C. After being washed with buffer 1 and precipitated by centrifugation at 150*g* for 1 min, the beads were boiled in SDS loading buffer and analyzed by SDS-PAGE and western blot detection using the in-house NS5 antibody 5M1 (Zhao et al. 2014).

### Dynamic light scattering

Dynamic light scattering (DLS) was used to generate the intensity-weighted size distribution of WT NS5, NS5 R888A mutant, 3′SL RNA, and 1:1 molar ratio mixture of WT NS5 or R888A with 3′SL. An amount of 15 µL of sample in DLS buffer (100 mM NaCl, 1 mM TCEP, 50 mM HEPES, pH 7.0) was loaded into a fluorescence quartz cuvette (Hellma) and measured by Zetasizer NanoS (Malvern) equipped with a backscatter detection system at 173° at 25°C. WT NS5 at 1.3 and 0.65 mg/mL, R888A mutant at 1.94 and 0.65 mg/mL, 3′SL RNA at 1.45 and 0.72 mg/mL were measured multiple times independently to eliminate concentration effect on the estimation of particle size. The hydrodynamic radius R_H_ of the particle which is defined as the radius of a sphere having the same diffusion rate as the particle was calculated by the Stokes–Einstein equation:

$$R_{H}=k_{B}T/6\pi \eta D,$$

where k_B_ is the Boltzmann coefficient, T is the absolute temperature, η is solvent viscosity, D is the diffusion coefficient which is determined from the auto-correlation function describing the fluctuation of scattered light. The scattering profile was analyzed by Zetasizer Nano software (version 6.01) using the non-negative least-squares algorithm to deconvolute the correlation curve to intensity-weighted size distribution.

### Statistical analysis

The statistical significance of difference between the two groups was evaluated using Student's *t*-test with two-tailed distribution and two-sample method assuming unequal variances. Two-way ANOVA with Bonferroni correction was performed to determine significant differences between groups for the intracellular and extracellular viral RNA kinetics data. *P*-values ≤0.0001, ≤0.001, ≤0.01, ≤0.05 were represented by [****], [***], [**], [*], respectively; *P*-values >0.05 were not considered statistically significant.

## SUPPLEMENTAL MATERIAL

Supplemental material is available for this article.

## Supplementary Material

Supplemental Material

## Figures and Tables

**Sai Wang:**
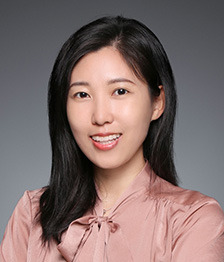


## MEET THE FIRST AUTHOR

***Meet the First Author(s)* is a new editorial feature within *RNA*, in which the first author(s) of research-based papers in each issue have the opportunity to introduce themselves and their work to readers of *RNA* and the RNA research community. Sai Wang is the first author of this paper, “A conserved arginine in NS5 binds genomic 3′ stem–loop RNA for primer-independent initiation of flavivirus RNA replication.” Sai did this work as a postdoctoral fellow in the laboratory of Subhash Vasudevan at Duke-NUS Medical School, National University of Singapore, working on dengue virus genomic RNA-related projects, and is currently a Senior Scientist in the Mammalian Protein Production platform in GenScript Biotech, Singapore.**

**What are the major results described in your paper and how do they impact this branch of the field?**

We identified a critical role for the R888 of DENV NS5 in binding to the 3′ stem–loop motif, a feature that is conserved among flaviviruses. Our paper has proposed a novel model for de novo initiation of DENV genomic RNA replication, through a coordinative network of NS5 bound 5′ SLA and 3′ SL juxtaposed by NS5 dimerization.

**What led you to study RNA or this aspect of RNA science?**

Before joining the Vasudevan laboratory, my research project was focusing on the structural characterization of the macromolecule of DNA and histone complex—chromatin and nucleosome. As the genetic information carrier, DNA wraps on and unwraps off from the histone octamer dynamically, modulating the gene transcription activities. Viruses like dengue virus utilize a single strand of the RNA molecule as genomic material. As compared to DNA, RNA molecules are more flexible and versatile. Viral RNA functions through dynamic interactions with viral and host proteins to accomplish viral replication and maintain viability. I was fascinated by this flexible, negatively charged string which circularizes, bends, and folds, forming various secondary and tertiary structural motifs like stem–loops, dumbbells, and pseudoknots. Its versatility reminds me of the Cat's cradle game I used to play as a little girl.

**During the course of these experiments, were there any surprising results or particular difficulties that altered your thinking and subsequent focus?**

The results leading to the novel model proposed in the paper were made possible because of the NS5 mutations that were tested in cells using infectious cDNA clones, as well as overexpressed and purified proteins. I made various deletions in the 3′SL RNA to further narrow down the sequence that interacted with NS5 R888 to confirm the specificity of interaction.

**If you were able to give one piece of advice to your younger self, what would that be?**

Be more confident and proactive and not afraid of making mistakes.

**Are there specific individuals or groups who have influenced your philosophy or approach to science?**

My PI Professor Subhash Vasudevan had a great influence, in a positive way, on my way to do research. From him, I learned the essence of being a good researcher—teamwork to maximize creativity and ensure efficiency, regular updates to keep track of project progress, and win-win collaboration to combine forces to reach a broader horizon and achieve a bigger goal. This philosophy is still working wonderfully in my current work environment as a Senior Scientist in GenScript Biotech.
