# Supplementary material for: A conserved arginine in NS5 binds genomic 3′ stem–loop RNA for primer-independent initiation of flavivirus RNA replication
Source: RNA. 2022 Feb;28(2):177–93. doi: 10.1261/rna.078949.121 (PMC8906541; doi:10.1261/rna.078949.121)
Supplement: Supplemental Material [file supp_28_2_177__DC1.html]

A conserved arginine in NS5 binds genomic 3′ stem–loop RNA for primer-independent initiation of flavivirus RNA replication — Supplemental Material 

# A conserved arginine in NS5 binds genomic 3′ stem–loop RNA for primer-independent initiation of flavivirus RNA replication

## Supplemental Material

- Supplemental\_Material.pdf
