# Supplementary material for: A conserved arginine in NS5 binds genomic 3′ stem–loop RNA for primer-independent initiation of flavivirus RNA replication
Source: RNA. 2022 Feb;28(2):177–93. doi: 10.1261/rna.078949.121 (PMC8906541; doi:10.1261/rna.078949.121)
Supplement: Supplemental Material [file supp_078949.121_Supplemental_Material.pdf]

A conserved arginine in NS5 binds genomic 3'stem-loop RNA for primer-independent initiation of flavivirus RNA-replication

Sai Wang[a], Kitti Wing Ki Chan[a], Min Jie Alvin Tan[a], Charlotte Flory[a], Dahai Luo[c], Julian Lescar[d], Jade K Forwood[e], Subhash G Vasudevan*[a,f,g]

Supplemental Material

Supplementary Figure 1

A

```
                                                  top-loop   side-loop
             DENV2 AGATCCT-GCTGTCT--CCTCAGCA--TCATTCCAGGCACAGAACGCCAG-AAAAT-GG-----AATGGTGCT--G--T-TGAA--TCAACAGGTTCT
             DENV1 AGATCCT-GCTGTCT--CTACAGCA--TCATTCCAGGCACAGAACGCCAG-AAAAT-GG-----AATGGTGCT--G--T-TGAA--TCAACAGGTTCT
             DENV3 AGATCCT-GCTGTCT--CCTCAGCA--TCATTCCAGGCACAGAACGCCAG-AAAAT-GG-----AATGGTGCT--G--T-TGAA--TCAACAGGTTC-
             DENV4 AGATCCT-GCTGTCT--CTGCAACA--TCAATCCAGGCACAGAGCGCCGC-GAGAT-GG-----ATTGGTGTT--G--T-TGAT--CCAACAGGTTCT
        ZIKV_MR766 AGACTCCATGAGTTT--CCACCACGCTGGCCGCCAGGCACAGATCGCCGA-ACAGC-GG-C--GGCCGGTGTG--G--G-GAAA--TCCATGGTTTCT
    ZIKV_H/PF/2013 AGACTCCATGAGTTT--CCACCACGCTGGCCGCCAGGCACAGATCGCCGA-ATAGC-GG-C--GGCCGGTGTG--G--G-GAAA--TCCATGGGTCT-
    ZIKV_Paraiba01 AGACTCCATGAGTTT--CCACCACGCTGGCCGCCAGGCACAGATCGCCGA-ATAGC-GG-C--GGCCGGTGTG--G--G-GAAA--TCCATGGGTCT-
               YFV AGTGGTTCTCTGCTT--TTCCTCCAGAGGTCTGTGAGCACAGTTTGCTCA-AGAAT-AA-GCAGACCTTTGGA--T--GAC--A--AACACAAAACCA
               WNV GGATCTTCTGCTCTG-----CACAACCAGCCACACGGCACAGTGCGCCGA-CATAG-GT----GGCTGGTGGT--G----CTAG--AACACAGGATCT
              MVEV AGATCTTCTGCTCTA--TTCCAACATCAGTCACAAGGCACCGAGCGCCGA-ACACT-GT----GACTGATGGG--G--GAGAAG--ACCACAGGATCT
              TBEV GGTTCTTGTTCTCCCTGAGCCACCATCA---CCCAGACACAGGTAGTCTG-ACAAG-GA-GGTGATGTGTGAC--T--CGGAAA--AACACCCGCT--
               JEV AGATCTTCTGCTCTA--TCTCAACATCAGCTACTAGGCACAGAGCGCCGA-AGTAT-GT----AGCTGGTGGT--G--AGGAAG--AACAC-------
 USUV_Vienna-2001 AGATCTTCTGCTCTA--TTCCAACATCAACCACAAGGCACAGAGCGCCGA-AAATT-GT----GGCTGGTGGG--G--AACTAG--ACCACAGGATCT
               BCV AAAGGTTCATTA------A-CTACCTGAGTGAAATCGGGAGGTACTCCGA-AGAAG-A-----GA-------------AGGCA------TTTGCCTT
              ENTV AGACCCCCCGCGCCCATAACCAACATAAA---------ACAG-------C-ATATT-G------ACACCTGG------GAAAG--ACCGGAGACTCT
              MMLV TGGATATATACT--------CCAGCC-AG--AAAAGACTCAGATTGTCTC-ATGACTT--TCTGACTGGC-G---T--ACATA---GCCATCCGCT--
              PPBV AAAGGTTCATTA------A-CTACTTGAGTGAAATCGGAAGGTATTCCGA-AAAAG-A-----GA-------------GGGCG------TTTGTCTT
               RBV AAAATTACGTCA------A-CTACCTTGGAGCAATGGGACGCTATGAATG-TGATG-A-----TG-------------TCTCA------TTCCAACT
              YOKV ------TCGGTTCCGGAGAGCTCCGGAGG--CCAGGGCGCCGTTTGCCCG-TAGTTTATAACTGGCCTTCGGGGATCGAAGGAGTTGCCAAACACT--
             APOIV AGCATTACTCTG------A-CTACTTGAGCAATATGGGTAGGTACCAAGA-AGGGA-C-----GG-------------AAGAG------TTCCACAT
              JUTV AAAACTTCAGGG------A-TTACCTTCAAGTGATGGGCAGGTTTGTTCA-AAAAC-A-----GCCTTCAG--------CCACC------TTCTCCAT
              MODV TGATTAGCCATG-----G--TCGCAC-AG--ATCAAGCTCAGATTGCTTACATG-TAA--TCTGTGTGGTCA---T--GAATA--TGACCTCCGCT--
```

B

```
                           838                                               888
            DENV2 VESWEEIP Y LGKREDQWCGSLIGLTSRATWAKNIQTAINQVRSLIGN-EEYTDYMPSMK R FRREEEEAGVLW--
            DENV1 ISSWEDVP Y LGKREDQWCGSLIGLTARATWATNIQVAINQVRRLIGN-ENYLDYMTSMK R FKNESDPEGALW--
            DENV3 VTTWENVP Y LGKREDQWCGSLIGLTSRATWAQNIPTAIQQVRSLIGN-EEFLDYMPSMK R FRKEEELEGAIW--
            DENV4 VHSWEDIP Y LGKREDLWCGSLIGLSSRATWAKNIHTAITQVRNLIGK-EEYVDYMPVMK R YSAPSESEGVL---
       ZIKV_MR766 VTKWTDIP Y LGKREDLWCGSLIGHRPRTTWAENIKDTVNMVRRIIGDEEKYMDYLSTQV R YLGEEGSTPGVL--
   ZIKV_H/PF/2013 VTKWTDIP Y LGKREDLWCGSLIGHRPRTTWAENIKNTVNMVRRIIGDEEKYMDYLSTQV R YLGEEGSTPGVL--
   ZIKV_Paraiba01 VTKWTDIP Y LGKREDLWCGSLIGHRPRTTWAENIKNTVNMVRRIIGDEEKYMDYLSTQV R YLGEEGSTPGVL--
              YFV VKKWRDVP Y LTKRQDKLCGSLIGMTNRATWASHIHLVIHRIRTLIGQ-EKYTDYLTVMD R YSVDADLQLGELI-
              WNV VERWSDVP Y SGKREDIWCGSLIGTRTRATWAENIHVAINQVRSVIGE-EKYVDYMSSLR R YEDTIVVEDTVL--
             MVEV VSDWTEVP Y VGKREDIWCGSLIGTRTRATWAENIYAAINQVRSVIGK-EKYVDYVQSLR R YEETHVSEDRVL--
             TBEV VMEWRDVP Y LPKAQDMLCSSLVGRRERAEWAKNIWGAVEKVRKMIGP-EKFKDYLSCMD R HDLHWELRLESSII
              JEV ITSWTDVP Y VGKREDIWCGSLIGTRSRATWAENIYAAINQVRAVIGK-ENYVDYMTSLR R YEDVLIQEDRVI--
 USUV_Vienna-2001 VQSWTDIP Y TGKREDIWCGSLIGTRTRATWAENIYAAINQVRAIIGQ-EKYRDYMLSLR R YEEVNVQEDRVL--
              BCV INDWKNIP Y LPRGQDISCGSLVGTGKRAQWAELIPGATLKVRNLMGN-ERFINYLSEIG R YSEEE--KAFALY-
             ENTV LEDWKEIP Y LNKSQDVRCGSHIGCSQRKSWADALPSTVEKVRSIIGKDGKYVDYMQTQT R FATIVRTMFGDVL-
             MMLV VREWNAIP Y LPRREDINCGSLIGTSKRSTWATLVPGAVMKVRNLFGP-EKFSNYMDCIG R YHVGH--QDFCLY-
             PPBV INDWKDVP Y LPRGQDISCGSLVGTGKRAQWAELIPGATLKVRNLMGN-ERFINYLSEIG R YSEKE--RAFVLY-
              RBV ITTWKNVA Y LPRGQDMCCGSLIGSSKRATWAKLIPGAVEKVRGMIGN-ENYVNYLGAMG R YECDD--VSFQLY-
             YOKV IGKWQDIP Y ISKSQDVRCGSMIGTSKRSSWAEALPHTVQKVRGIVGTQERYRDYLETQN R FRTIVQHLVGDIL-
            APOIV VAEWKNIP Y LQRNQDLSCSSLIDNPTRAQWAKLLKGAVMKVREMIGR-EHYSDYLSNMG R YQEGT--EEFHMW-
             JUTV IDQWSNIP Y LPRKVDKKCGSLIGMKNRIEWAKLLPGAVLKVRNVFGR-ENFRDYLQVMG R FVQKQPSATFSMY-
             MODV VERWSEVP Y LPRNQDKSCGSLIGTTARAEWAKLLPGAVEKVRNIFGK-QRFRNYLRNMG R YESQE-EAPFSMY-
```

Supplementary Figure 1. Multiple sequence alignment of the (A) 3'SL and (B) NS5 C-terminal sequences of flaviviruses. The residues Y838 and R888 are highlighted in bold in (B).
GenBank accession numbers for the relevant sequences are: DENV1 (EU081230), DENV2 (EU081177), DENV3 (EU081190), DENV4 (GQ398256), ZIKV_MR766 (LC002520.1), ZIKV_H/PF/2013 (KJ776791.2), ZIKV_Paraiba01/2015 (KX280026.1), Yellow Fever virus (YFV; X15062), West Nile virus (WNV; M12294), Murray Valley Encephalitis virus (MVE; AF161266), Tick-Borne Encephalitis Virus (TBEV; U27495), Japanese Encephalitis virus (JEV; M55506), Usutu virus (USUV_Vienna-2001; AY453411), Batu Cave Virus, strain P70-1459 (BCV; KJ469370.1), Entebbe bat virus, strain UgIL-30 (ENTV; NC_008718.1), Montana myotis leukoencephalitis virus, stain UNKNOWN-NC_004119 (MMLV; NC_004119.1), Phnom Penh bat virus, strain 30834_A38 (PPBV; NC_034007.1), Rio Bravo virus, strain RiMAR (RBV; NC_003675.1), Yokose virus, strain Oita 36 (YOKV; NC_005039.1), Apoi virus, strain ApMAR (APOIV; NC_003676.1), Jutiapa virus, strain JG-128 (JUTV; NC_026620.1), Modoc virus, strain M544 (MODV; NC_003635.1).

Supplementary Figure 2

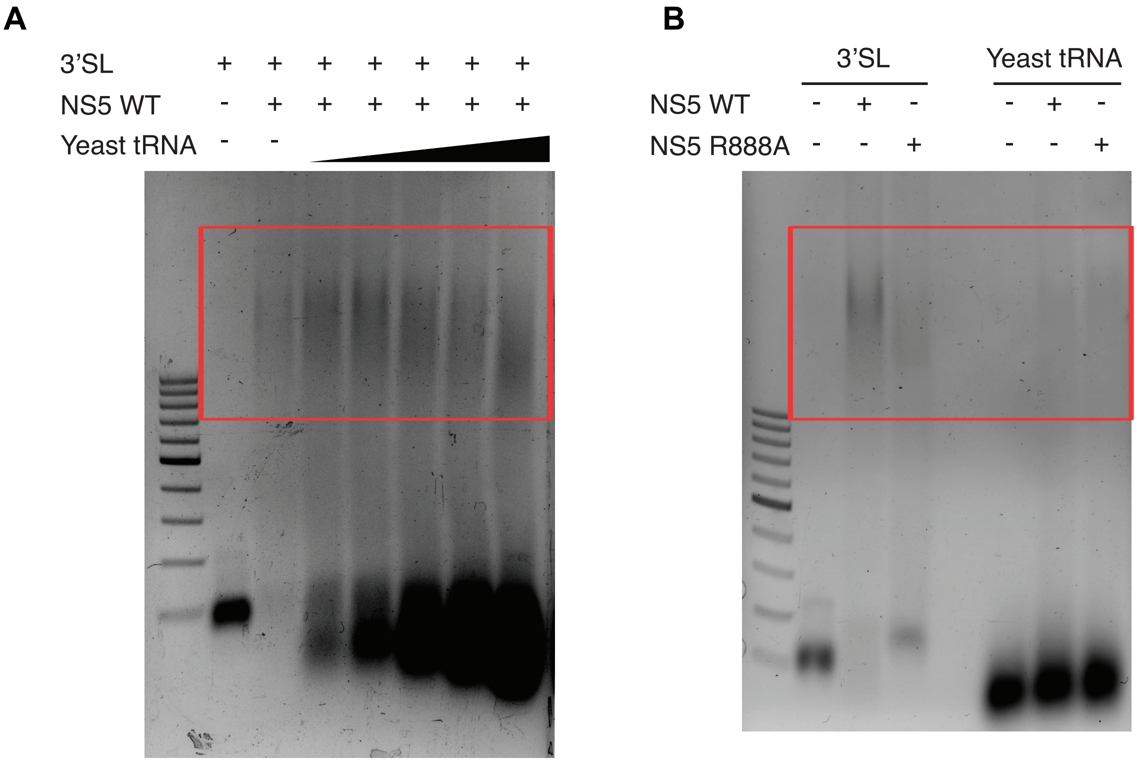

Supplementary Figure 2. 3' SL binds specifically to wild type (WT) DENV2 NS5. (A) 3'SL was incubated with DENV2 NS5 in the absence and presence of increasing amounts of yeast tRNA (0.1, 0.5, 1, 2 and 5ug). (B) 3'SL or yeast tRNA was incubated with wild type and R888A DENV2 NS5. In both panels, the reactions were resolved by agarose gel electrophoresis and the RNA was detected using GelRed. An interaction between the RNA and NS5 protein will result in a shift in the RNA from the bottom of the gel to the top (area boxed in red)

Supplementary Figure 3

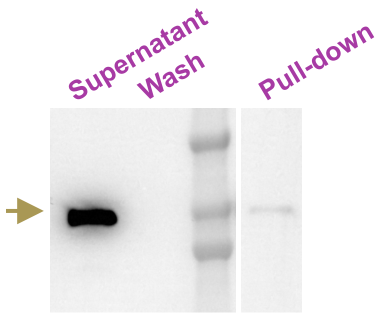

Supplementary Figure 3. Western blot analysis of various samples (supernatant from centrifugation following the incubation of NS5 R888A protein with beads; wash of beads after centrifugation and removal of supernatant; pull-down) from NS5 R888A mutant and biotinylated 5'SLA RNA Co-IP experiment. NS5 was detected by 5M1 antibody.

Supplementary Figure 4

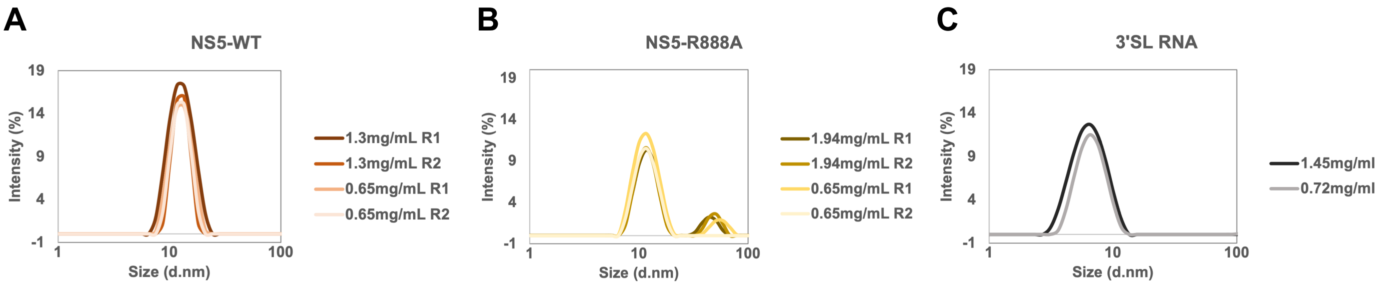

Supplementary Figure 4. Intensity-based size distribution profiles from Dynamic Light Scattering (DLS) experiments showing **[A]** WT NS5 at two different concentrations and each with two technical repeats; **[B]** NS5 R888A mutant at two different concentrations and each with two technical repeats and **[C]** 3'SL RNA at two different concentrations**.** X-axis represents hydrodynamic diameter of NS5 or 3'SL RNA molecules. Concentrations of NS5 or 3'SL RNA are noted in the legend of each sub-figure.

Supplementary Figure 5

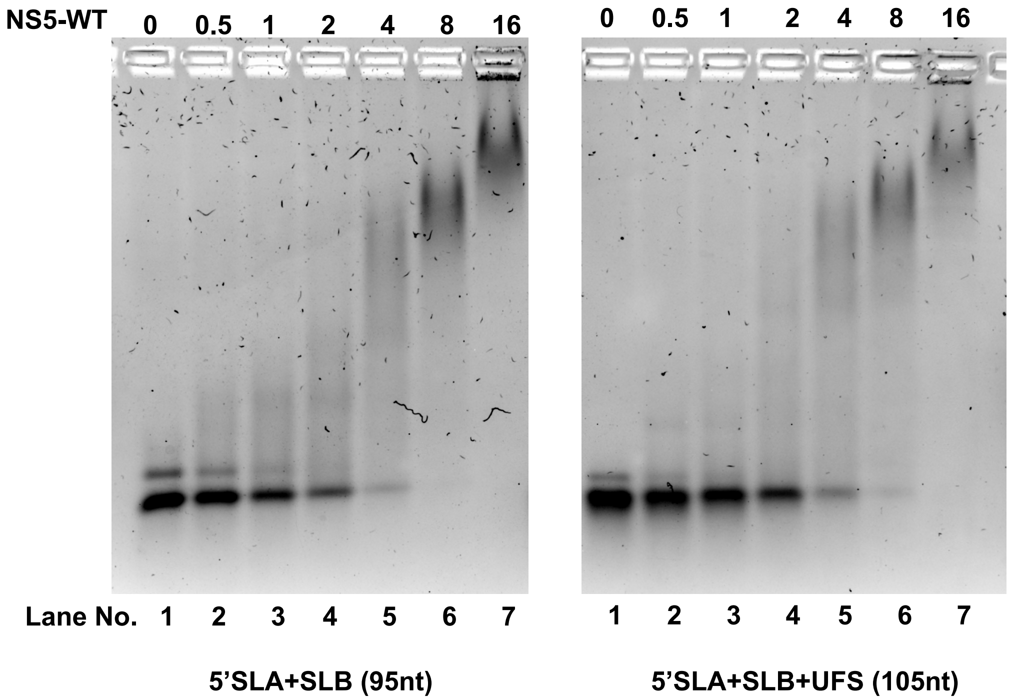

Supplementary Figure 5. RNA electrophoretic mobility shift assay (REMSA) of 5’ SLA+SLB or 5’SLA+SLB+UFS with WT NS5 that was analyzed by agarose gel electrophoresis and visualized by GelRed staining. Lane 1 in each gel represents 5’ SLA+SLB or 5’SLA+SLB+UFS RNA alone in binding buffer. Lanes 2-7 represents binding reactions of NS5 with RNA at molar ratio of 0:1, 0.5:1, 1:1, 2:1, 4:1, 8:1 and 16:1 as indicated at the top of each lane.
